# Biosensor for Bacterial Detection Through Color Change in Culture Medium

**DOI:** 10.3390/bios15080551

**Published:** 2025-08-20

**Authors:** Aramis A. Sánchez, Grettel Riofrío, Darwin Castillo, J. P. Padilla-Martínez, Vasudevan Lakshminarayanan

**Affiliations:** 1 PROSUR Construcción Sustentable del Sur, Puebla 72810, Mexico; gariofrio1@utpl.edu.ec; 2 Department of Chemistry, Universidad Técnica Particular de Loja, Loja 1101608, Ecuador; 3 Theoretical and Experimental Epistemology Lab, School of Optometry and Vision Science, University of Waterloo, Waterloo, ON N2L 3G1, Canada; vengulak@uwaterloo.ca; 4 Institute of Sciences, Benemérita Universidad Autónoma de Puebla, Puebla 72960, Mexico; juan.padilla@correo.buap.mx; 5 Departments of Physics, Electrical and Computer Engineering and Systems Design Engineering, University of Waterloo, Waterloo, ON N2L 3G1, Canada

**Keywords:** optical detection, optic biosensor, *Staphylococcus aureus*, colorimetric sensing, culture medium

## Abstract

Rapid and accurate bacterial detection is essential in medicine, the food industry, and environmental monitoring. This work presents the development of an optical sensor based on color changes in the culture medium that leverages the optical interaction of bacterial metabolic products. The proposed prototype operates on the principle of optical transmittance through mannitol salt agar (ASM), a selective medium for *Staphylococcus aureus*. As bacterial growth progresses, the medium undergoes changes in thickness and, primarily, color, which is optically measurable at specific wavelengths depending on the type of illumination provided by the simplified light-emitting diodes (LEDs). The sensor demonstrated the ability to detect bacterial growth in approximately 90–120 min, offering a significant reduction in detection time compared to traditional incubation methods. The system is characterized by its simplicity, sensitivity, low reagent consumption (up to 140 fewer reagents per test), and potential for real-time monitoring. These findings support the viability of the proposed sensor as an efficient alternative for early pathogen detection in both clinical and industrial applications. Finally, a proposal for simplifying the sensor in a system composed of a light-emitting diode and a light-dependent resistor is presented.

## 1. Introduction

The efficient detection of pathogenic microorganisms is essential in various fields. According to the World Health Organization (WHO) [1], the global burden of infectious diseases is at its highest, especially in low- and middle-income countries. Just one year before the COVID-19 pandemic, they were responsible for about 704 million disability-adjusted life years (DALYs) associated with 85 pathogens worldwide, with bacterial infections contributing ~415 million DALYs, viral infections ~178 million, and parasitic diseases ~172 million [2,3].

Recent estimates [1,2,3,4] indicate that bacterial infections were responsible for approximately 7.7 million deaths in 2019, accounting for approximately 14% of deaths globally, making them the second leading cause of death worldwide after ischemic heart disease. Among 33 bacterial pathogens analyzed by [4], it was determined that five alone, *Staphylococcus aureus*, *Escherichia coli*, *Streptococcus pneumoniae*, *Klebsiella pneumoniae*, and *Pseudomonas aeruginosa*, are responsible for more than half of these deaths.

In this sense, the detection of pathogenic bacteria in clinical, food, or environmental samples is essential for infection control, outbreak prevention, and food safety assurance. The traditional methods based on microbial culture, while effective, require long incubation periods and specialized laboratories. Instead, optical sensors offer rapid, portable, and specific detection, making them a promising solution for on-site applications [5,6].

In this context, optical sensors have revolutionized diagnostic microbiology by offering viable alternatives to traditional culture methods, which, while specific, typically require 24 to 72 h to obtain results [7].

Optical sensors identify bacterial presence by detecting variations in optical parameters, such as absorbance, transmittance, reflectance, intensity, phase, polarization, wavelength, refractive index, fluorescence, or visible color change, triggered by microbial metabolism [8]. These sensors are known for their high sensitivity, rapid detection capacity, compact design, and, in many cases, portability [9,10,11]. The combination of this technology with colorimetric detection, where sensors respond to visible optical transformation associated with biochemical processes, such as environmental acidification or the degradation of indicator compounds, enables the development of low-cost, highly sensitive, and visually interpretable devices [12,13].

In this article, we present a novel method for the detection of *Staphylococcus aureus* using an optical setup that detects changes in transmittance intensity at four different wavelengths and correlates these changes with the presence of *Staphylococcus aureus*.

## 2. Related Work and Theoretical Framework

### 2.1. Physical Principles of Optical Sensors for Detection

Optical sensors operate using photometry, spectroscopy, or interferometry. Essentially, a light source (e.g., an LED or tungsten halogen lamp) emits light through a sample contained in an optically accessible medium. A detector (photodiode, spectrometer, charge-coupled device (CCD), or digital camera) captures the light signal transmitted or reflected by the sample, quantifying changes over time or at specific wavelengths [14].

Key parameters include the following: Wavelength range: typically, within the visible spectrum (400–700 nm) or UV-Vis for higher spectral resolution. Interaction type: direct transmittance, differential absorbance, or diffuse reflectance. Temporal resolution: from seconds to several hours. Optical configuration: linear configuration, fiber optic, integrated spectroscopy, or digital imaging [15].

### 2.2. Optical Biosensors: Principles and Advantages

Fiber optic biosensors can be classified according to their detection principle. Intensity-based sensors are the simplest and most robust, as they detect the attenuation or amplification of light transmitted through the fiber due to optical interactions with the surrounding medium. This type of sensor has been used to monitor bacterial growth in real time by tracking changes in the refractive index of the medium, as demonstrated by Soares et al. [16] with *E. coli* in a liquid medium.

More sophisticated approaches utilize phenomena such as surface plasmon resonance (SPR), in which an electromagnetic wave resonates with electrons on the surface of a metal-coated fiber, altering the optical signal in the presence of specific bacteria or biomarkers [17,18]. These sensors have demonstrated high sensitivity in complex matrices such as food and biological fluids.

In practice, optical biosensors offer multiple advantages over conventional methods: they allow for online analysis, are compatible with hostile or contaminated environments, do not necessarily require additional biological markers, and can be easily miniaturized for point-of-care applications [19]. Small and harmless electrodes have also been added for bacterial culture, reducing the size of the reactors and facilitating their deeper analysis [20], such as those proposed by Lopez-Buenafe [21] made from a transparent PDOT: PSS electrode.

### 2.3. Optical Sensors and Colorimetry in Culture Media

In microbiological applications, colorimetry is a widely used strategy to infer bacterial presence or activity based on visible changes in the color of the culture medium. These changes can be associated with carbohydrate metabolism (e.g., glucose fermentation); the production of acids, ammonia, or phenolic compounds; as well as the alteration of redox or pH-sensitive indicators [19]. Innovative systems have leveraged these biochemical principles along with optical platforms to develop portable, low-cost sensors that do not require complex instrumentation [22,23,24].

Bunin et al. (2023) [5] provide a comprehensive review of optical sensors for bacterial detection, highlighting systems based on colorimetry and fluorescence. They argue that the combination of environmentally sensitive materials with optical systems enables ultra-low detection limits, even in the presence of interferents [5]. Similarly, Idil et al. [6] highlight that the integration of nanomaterials, such as quantum dots, metallic nanoparticles, or molecularly imprinted polymers, significantly improves the selectivity and spectral response of these sensors.

In a direct experimental approach, Zhang et al. [12] developed a nanofiber-based colorimetric platform for the detection of *Escherichia coli* in clinical and food matrices, achieving a response time of less than 20 min with visual detection that does not require readout devices.

Beyond bacterial culture, as indicated by [20], optical/electrochemical modules are already used for in vitro cell monitoring (DO, pH, temperature, and metabolites) in flasks, microfluidic chips, and bioreactors, enabling non-invasive, real-time readouts with fiber optic probes and luminescent films (e.g., PtTFPP 405/650 nm), as well as in situ imaging (OCT/LSPR) and impedance arrays for confluence/migration.

### 2.4. Colorimetric Detection of Bacterial Byproducts

An alternative approach to direct bacterial detection is to identify metabolic products that cause optical changes in the medium. For example, several studies have incorporated biomimetic membranes or reactive matrices containing tetrazolium salts or redox indicators that undergo visible color changes when reduced by active bacterial enzymes [13,25]. This type of visual signal can be quantitatively correlated with bacterial concentration and, therefore, used as a reliable indicator of microbial contamination. Zhou et al. [17] demonstrated a technique based on glucose metabolism in which bacterial activity enzymatically transforms glucose, resulting in a measurable color change implemented on a paper-based reagent matrix [20,26,27]. These systems are particularly attractive due to their low cost, portability, and adaptability.

Designing an effective optical sensor also requires careful consideration of the optical properties of the culture medium, as this can absorb, scatter, or interfere with the optical signal. In vitro experiments have shown that selecting buffers and media with low absorbance in the visible or infrared range improves the signal-to-noise ratio of the sensor system [28,29]. Some of them are used in food analysis [30] and in different thin-film deposition techniques [31]. Furthermore, sensors incorporating porous structures or polymers with affinity for bacterial compounds allow for a closer interaction between the optical fiber and the analyte [32,33,34].

In research conducted by Vázquez et al. (2006) [13], biomimetic membranes visibly reacted with substances secreted by bacteria such as *Pseudomonas* and *E. coli*, allowing for clear chromatic differentiation between species.

Correspondingly, in [21] it is shown that bioelectronic interfaces based on PEDOT: PSS microstructured electrodes fabricated by vacuum soft lithography on PDA-functionalized ITO achieve faithful geometry replication (~222–417 µm width; ~2.8–3.3 µm thickness), optimal electrochemical performance at 2% GOPS (low impedance), and cytocompatibility with MDA-MB-231 cells (XTT, Live/Dead); BSA (0.1%) confines cell adhesion to the PEDOT: PSS active region.

### 2.5. Types of Optical Sensors for Bacterial Detection

Transmittance-based sensors: These sensors quantify the amount of light passing through a culture medium. Cushla et al. (2021) [35] developed a system using a halogen–tungsten light source and a photodetector inside a sealed vial containing ASM, achieving *Staphylococcus aureus* detection in approximately 150 min.

RGB imaging and machine vision: Imaging systems capture periodic photographs of the culture surface and analyze them using RGB algorithms. Romphosri et al. (2024) [36] applied machine learning to RGB image data to differentiate colonies of *Staphylococcus aureus, E. coli*, and others with high accuracy.

Fiber optic sensors use light transported through fibers to detect changes in the refractive index or intensity of transmitted light due to microbial activity. These systems are suitable for in situ applications, as they can be integrated into fluidic environments. Pendão and Silva (2022) [14] reported on highly miniaturized and sensitive fiber optic biosensors.

LED and photodiode modules are compact devices that integrate light-emitting diodes and photodetectors. Wildeboer et al. (2010) [37] designed a system to detect *Staphylococcus aureus* based on changes in light intensity, achieving detection limits below 10^3^ CFU/mL in less than 2 h.

A comparison of the detection range, media, light sources, and sensor types is shown in the accompanying table [36,38,39].

### 2.6. Mannitol Salt Agar and Staphylococcus aureus Detection

Mannitol salt agar (ASM) is a widely used selective and differential medium for isolating *Staphylococcus aureus*. It contains a high concentration of NaCl (7.5%), which inhibits the growth of non-halotolerant organisms, and mannitol as a fermentable carbohydrate. *Staphylococcus aureus* ferments mannitol, producing acid that lowers the pH and causes the phenol red indicator to turn from red to yellow [40].

This chromogenic change, easily visible to the naked eye, is ideal for quantitative optical detection, allowing for the design of devices that detect the presence of *Staphylococcus aureus* significantly faster than conventional culture methods [37].

## 3. Materials and Methods

### 3.1. Bacterial Strain Activation

The bacterial strain *Staphylococcus aureus* was supplied in cryopreserved format by the Microbiology Laboratory of Universidad Técnica Particular de Loja (UTPL). For reactivation, a single cryo-bead containing viable bacteria was aseptically transferred to a nutrient-rich tryptic soy agar (TSA) plate and incubated at 37 °C for 24 h to ensure colony development and metabolic reactivation. This method aligns with standard microbiological practices for recovering and cultivating dormant bacterial strains prior to experimental procedures [40].

The TSA medium serves as a general-purpose agar that supports the proliferation of most facultative anaerobes, including *Staphylococcus aureus*, and is routinely used as a preparatory step before inoculation in selective or differential media such as ASM [37,40].

### 3.2. Inoculation Protocols

After incubation, a bacterial suspension was prepared by emulsifying isolated colonies into sterile saline solution until a turbidity equivalent to 0.5 McFarland standard was achieved. Using an aseptic technique, 50 µL of the suspension was evenly distributed onto the surface of previously prepared mannitol salt agar (ASM) plates. These were then incubated at 37 °C for 24 h to allow for visible colony formation and metabolic activity—specifically mannitol fermentation, which leads to a characteristic color change in the medium due to acidification [40].

ASM is a selective and differential medium that inhibits non-halotolerant organisms due to its high NaCl concentration (7.5%) and visually indicates fermentation activity through phenol red pH indicator, which shifts from red to yellow upon acid production [37,40].

### 3.3. Prototype Design

The sensor prototype was designed to reduce reagent consumption and contamination risk while maintaining optical clarity and reproducibility. A standard transparent polypropylene microcentrifuge vial of 2 mL capacity was selected due to its mechanical stability, chemical inertness, and optical properties in the visible range. A small volume of 250 µL ASM was poured and solidified inside the inner surface of the vial cap, thus creating a minimal culture interface. The vial was hermetically sealed to prevent environmental contamination and moisture loss, ensuring consistent incubation conditions over the testing period.

**Figure 1:**
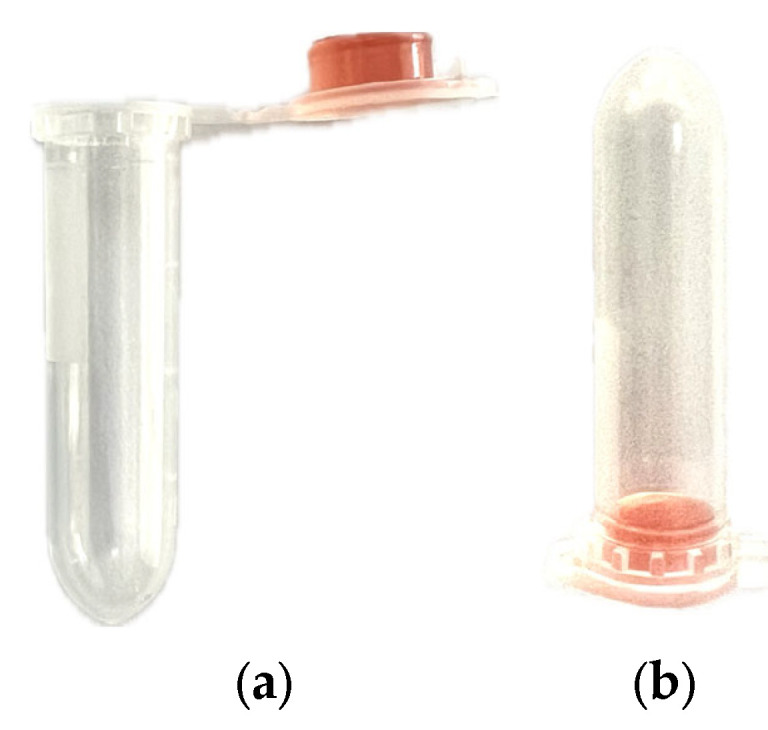
Prototype configuration showing 250 µL of mannitol salt agar contained in the cap of a 2 mL transparent polypropylene vial: (**a**) open, (**b**) closed.

This compact format achieved a 125-fold reduction in agar usage compared to traditional Petri dish methods [16] and allowed for stable optical alignment due to its fixed geometry (Figure 1). Moreover, the sealed environment ensured biosafety and allowed for the sample to be manipulated or transported without exposing laboratory personnel to the bacterial agent.

### 3.4. Optical Sensor Assembly

**Figure 2:**
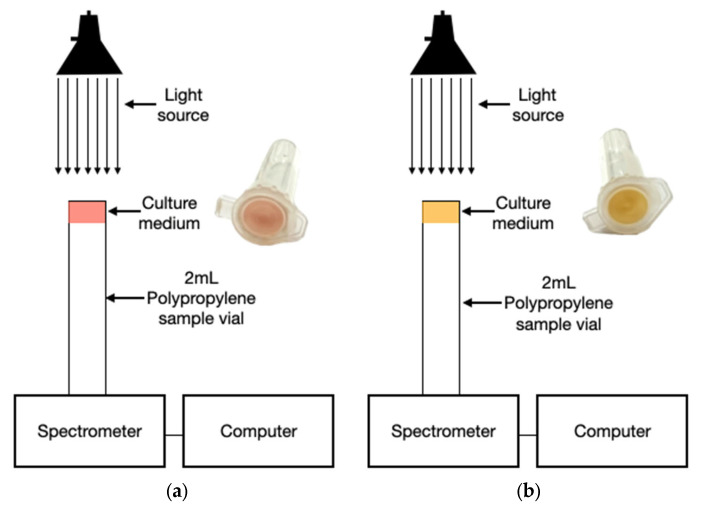
Optical detection system during incubation: (**a**) inoculated ASM with initial red coloration (pH > 7.4); (**b**) yellow coloration indicating acidification from mannitol fermentation (pH < 6.8).

The optical detection system was designed to measure the transmittance spectrum of the inoculated ASM medium over time. The experimental setup consisted of a halogen–tungsten light source (Thorlabs SLS201/M, Newton, NJ, USA), which provides stable broadband illumination from 360 to 2600 nm, and a UV-VIS spectrophotometer (Thorlabs CCS200, Newton, NJ, USA), capable of detecting spectral intensity across the 190–1020 nm range. The agar-containing vial was positioned precisely between the light source and the detector, allowing for the beam to pass directly through the medium (Figure 2a,b).

The optical signal collected by the spectrophotometer was continuously sent to a computer via USB interface, and real-time intensity spectra were recorded at regular intervals during the 24-h incubation period. Although full-spectrum data were available, the analysis focused on four principal wavelengths, 530 nm (green), 570 nm (yellow), 600 nm (orange), and 650 nm (red), which correspond to perceptible changes in the phenol red indicator during acidification and are compatible with commercially available monochromatic LED light sources [35,39].

The selected wavelengths (530–650 nm) match the phenol red absorbance range, especially around 570 nm, where pH-induced color change is maximal. The exclusion of blue and ultraviolet light from the analysis for a possible proposal for a blue or ultraviolet LED is because previous experiments showed that these wavelengths affect the incubation time of the bacteria, making this process slower, which is the opposite of what we want to achieve.

This transmission-based system is consistent with prior optical biosensor strategies for bacterial detection that utilize intensity variation as an indicator of metabolic activity [38,39]. However, unlike more complex imaging-based or interferometric setups [10], this system relies on a linear and reproducible configuration that facilitates signal acquisition and minimizes optical misalignment, and to avoid any type of light pollution, the experiment is carried out in a dark chamber. In this way, 50 test runs were carried out to check the reproducibility of the data and characterization of the sensor.

The real-time spectral acquisition enables non-destructive monitoring of bacterial growth dynamics and acid production. In this configuration, the sensor does not rely on colony morphology or physical expansion but instead captures biochemical changes in the medium, thus allowing for early detection before visual cues are noticeable to the naked eye [38].

This prototype design bridges the gap between conventional agar-based detection and high-cost, precision optical sensors by maintaining the accessibility and interpretability of colorimetric culture methods while incorporating a quantitative, automated optical readout. It lays the foundation for future simplification, adding a light-dependent resistor (LDR) into a fully LED-LDR-based analog detection system as proposed in subsequent sections [35].

## 4. Results

### 4.1. Sensor Performance

The polypropylene vial inevitably interacts with light; however, only a loss of intensity occurs since polypropylene absorbs in the infrared, and our research is carried out in a range of 190 nm to 1090 nm.

**Figure 3:**
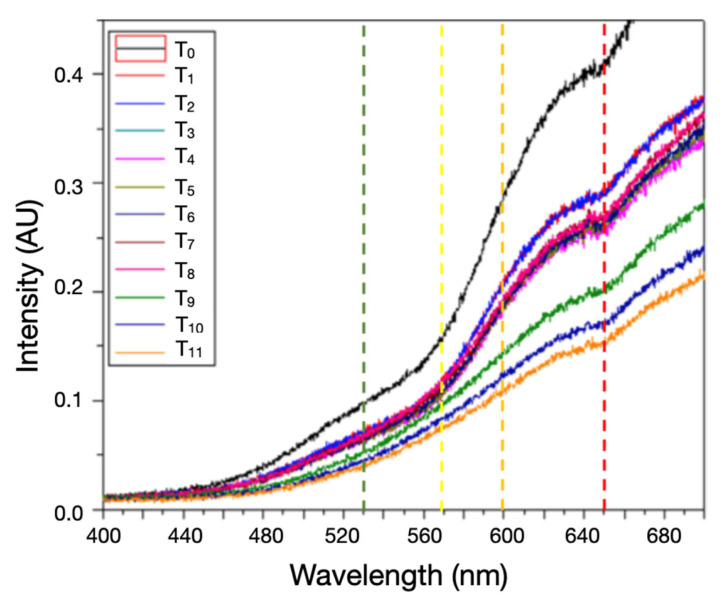
Spectral transmittance (400–700 nm) at 12 time points over 330 min (T_0_‒T_11_ = 0, 10, 20, 30, 60, 90, 110, 125, 140, 270, 305, 330 min; AU). Colored dashed lines indicate the four discrete bands corresponding to the wavelengths of the colors green (530 nm), yellow (570 nm), orange (600 nm), and red (650 nm). The progressive rise at 600–650 nm after ~110–150 min reflects the pH-dependent phenol-red shift during mannitol fermentation.

The prototype showed successful detection of *Staphylococcus aureus* growth within approximately 150 min. A visible color change in the ASM, from red to yellow, was evident, indicating mannitol fermentation and acid production. The intensity of the transmitted light shows a change over time, which is clearly seen in Figure 3; the light intensities are analyzed in the 400–700 nm window at the four positions marked with dotted lines, corresponding to the wavelengths of the colors green (530 nm), yellow (570 nm), orange (600 nm), and red (650 nm).

**Figure 4:**
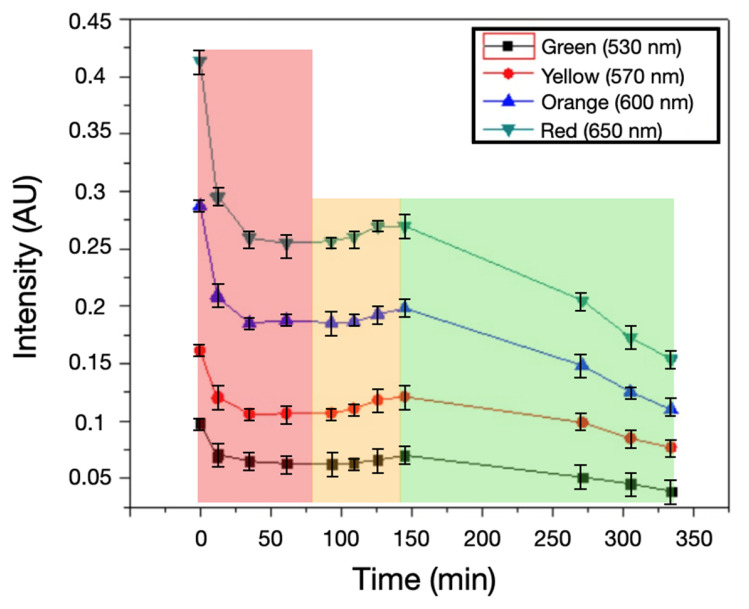
Time course of transmitted intensity at ■ 530 nm, 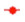 570 nm, 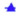 600 nm, and 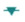 650 nm over 330 min (AU; mean ± SD). Background shading marks the regimes times period discussed in Section 4.2: red, 0–60 min—early attenuation (agar dehydration/thickness change and darkening); amber, 60–110 min—quasi-steady plateau; green, ≥130 min—detection window linked to mannitol-driven acidification (phenol-red shift), showing a peak around ~150 min followed by a gradual decline.

Spectral transmission profiles with *Staphylococcus aureus* over time showed an initial decrease in light intensity, followed by a constant light intensity in the 60–100 nm zone. After this, an increase in light intensity is present until it reaches a peak around 150 min, followed by a gradual decrease. The peak correlates with maximum yellow coloration, prior to bacterial overgrowth attenuating transmission (Figure 4).

**Figure 5:**
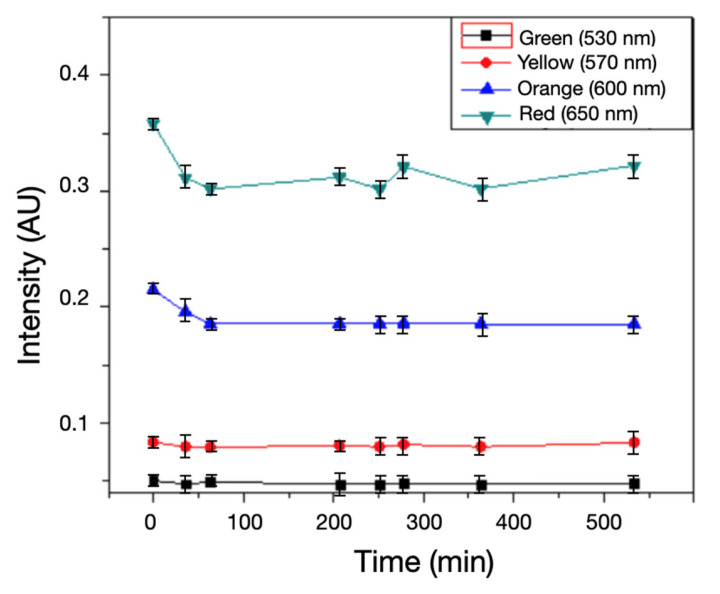
Uninoculated control: transmitted intensity at ■ 530 nm, 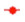 570 nm, 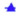 600 nm, and 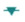 650 nm over ~520 min (mean ± SD; AU). All channels show a small initial decrease followed by stable levels, and notably no late-stage rise at 600–650 nm. This contrasts with inoculated samples (Fig. 4) and confirms that the signal increase is driven by bacterial metabolism, not instrument drift or illumination variability.

Regarding the uninoculated references, from Figure 5, it is clearly seen that for the selected wavelengths, only a decrease in the light transmission intensity is observed at the beginning, and then a constant transmission is maintained; this initial decrease may be due to the heat generated by the light source during the observation time.

The behavior of the transmitted light in the uninoculated sample (Figure 5) shows a slight variation at the beginning, within the first 50 min; this is mainly due to the change in morphology of the agar; the lamp is generating this change because the ASM in these quantities and under this lamp can be affected by the heat transferred by the lamp. This problem is solved by changing the light source to an LED, which is proposed by defining the best wavelength for the analysis.

### 4.2. Interpretation of Optical Signals

At t_0_, all wavelengths showed an initial drop due to agar dehydration, thickness reduction, and darkening of the ASM in the inoculated samples, all of them in the first 60 min (Figure 4 and Figure 5).

According to these variations and the Beer–Lambert law, there are two factors that determine the optical signal we obtain in the sensor, the change in the thickness of the sample and the change in color, which directly affect the absorption coefficient. This results in the behavior of the graphs in Figure 3 and Figure 4.

(1) $$I(\lambda ,l)=I_{0}e^{-\alpha (\lambda )l}$$

where I(λ,l) is the intensity transmitted through the sample, $I_{0}$ is the intensity of the light incident on the sample, α(λ) is the absorption coefficient dependent on the wavelength, and l is the thickness of the sample, which varies slightly due to the temperature produced by the lamp, a factor that can be fixed by using an LED light source.

There is a period of approximately 50 min in the 60–110-min zone in which the intensity remains constant for the inoculated samples (Figure 4). As for the uninoculated control samples, this intensity remains constant from 60 min and continues throughout the observation time (Figure 5).

In the window of 110 to 150 min, the intensity of the transmitted light increases because the ASM changes color to yellow, which allows for more light to pass through and reaches a maximum transmittance around 150 min (Figure 4).

After 150 min, the intensity of the transmitted light begins to decrease because the growth of bacterial colonies on the surface of the ASM begins to reflect more light (Figure 4). These optical patterns are consistent across the four selected wavelengths, with more pronounced changes observed at 600 nm (yellow) and 650 nm (red).

**Figure 6:**
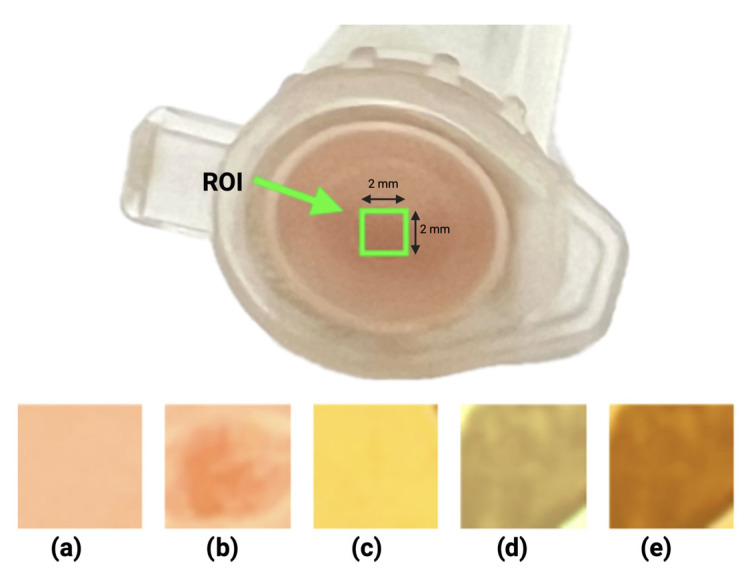
Evolution of transmitted light through the inoculated ASM sample in a vial cap under observation in an enclosed area. The ROI (region of interest, green square 2 × 2 mm) indicates the region used for the color/intensity analysis across stages (**a**–**e**). (**a**) Initial red color, (**b**) attenuation of transmitted light, (**c**) ASM color change, (**d**) and (**e**) gradual decrease in transmitted light.

The initial red zone in Figure 4 with high transmission intensity decreases due to a darkening of the ASM with the inoculated sample, which we see in the step from (a) to (b) in Figure 6; this is followed by a stable zone where the intensity of transmitted light does not change; then, it follows a color change from red to yellow that occurs until reaching an intensity peak around 150 min, which can be seen in the yellow zone in Figure 4 and (c) in Figure 6. Finally, when the bacterial colonies begin to cover the surface of the ASM, they begin to gradually prevent the passage of light, which is shown in the green zone in Figure 4 and (d), (e) in Figure 6.

The optical sensor system developed in this study demonstrates remarkable performance in terms of sensitivity, response time, and practicality when compared to traditional microbiological methods and more complex biosensing alternatives. The system effectively detects the presence of *Staphylococcus aureus* through the color change of mannitol salt agar (ASM), achieving positive detection in approximately 2–2.5 h. This represents a 10-fold reduction in detection time compared to the conventional 24-h incubation and observation required in standard culture-based methods [40].

A key advantage of the system lies in its low reagent consumption. While standard Petri dishes typically require 20–40 mL of agar per assay, this device operates with only 250 µL per test, enabling a reduction in reactant use by at least 80 times. This not only translates to significant cost savings but also aligns with sustainable laboratory practices, an increasingly relevant concern in global health systems [15,38].

In contrast to molecular detection methods such as polymerase chain reaction (PCR) or nucleic acid amplification tests (NAATs), this optical sensor setup does not require sample lysis, nucleic acid extraction, thermal cycling, or expensive probes or enzymes. These characteristics, while delivering excellent specificity in PCR, greatly increase the cost and complexity of diagnostic infrastructure [7,9]. The current device eliminates these bottlenecks by relying entirely on observable metabolic changes (i.e., acid production from mannitol fermentation) to trigger an optical signal, thereby preserving simplicity and accessibility.

While other optical systems, such as those using spectrophotometry [29], fiber optic interferometry [14], or RGB image classification [36,39], have also achieved reduced detection times, these often require costly and delicate components (e.g., spectrometers, optical filters, high-resolution cameras, and computing units). Moreover, these setups can demand controlled lighting and precise positioning, which may not be feasible in field or decentralized settings [39,41].

The present work circumvents these challenges by adopting a structural innovation: rather than introducing new reagents or consumables, the detection system was miniaturized and optimized through changes in geometry and light path configuration. This structural shift preserves the biological basis of detection (color change in ASM) but allows for greater reproducibility, alignment tolerance, and potential for automation or parallel testing.

**Figure 7:**
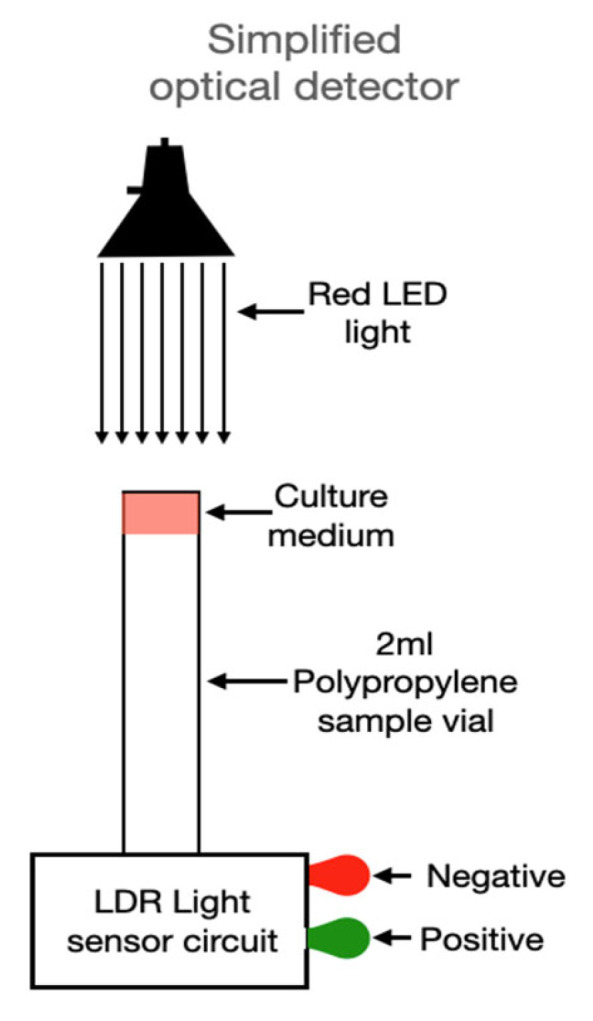
Simplified detector setup using a monochromatic light source and an LDR sensor to measure transmittance variation and identify *Staphylococcus aureus* growth in ASM through automated signal detection.

In its initial configuration, the device used a halogen–tungsten light source coupled with a spectrophotometer to quantify the transmittance change at specific wavelengths. Although accurate, this setup poses limitations for widespread use due to cost and bulk. However, based on the data obtained, a simplified version is proposed (Figure 7), replacing the broadband light source with a red LED (650 nm), selected to match the absorbance shift of the phenol red indicator used in ASM. In place of a spectrophotometer, a light-dependent resistor (LDR) is used as an intensity detector. This sensor, connected to a basic analog circuit, takes a single measurement after 90 min and then monitors for consistent intensity increases over a 10–20-min window. If a defined threshold is crossed, the system activates an optical or digital signal indicating the presence of *Staphylococcus aureus*.

This simplified architecture holds significant promise for mass deployment. Notably, the design can be manufactured using low-cost materials, powered by batteries or solar sources, and embedded in mobile or wearable diagnostic units. The detection logic is binary and does not require software interpretation, enabling use by non-specialized personnel—a critical requirement for deployment in remote or under-resourced areas.

When compared to fiber optic sensors described by Pendão and Silva (2022) [14], which require delicate fiber tips and often show drift or fouling over time, this system offers greater robustness and lower maintenance. Similarly, when contrasted with the AI-enhanced imaging models of Romphosri et al. [36], which provide high classification performance but demand camera calibration and processing hardware, this sensor stands out in portability and immediacy of results.

Furthermore, unlike surface plasmon resonance sensors [40], which require metal coatings and nanostructured surfaces for refractive index modulation, the proposed system uses standard, transparent plastic or glass containers and does not rely on any surface engineering or immobilization chemistry, dramatically reducing barriers to implementation.

Importantly, the design maintains biosafety by allowing for the sample to remain sealed throughout the detection process. This feature protects the environment and the operator from potential contamination, which is particularly relevant in settings where bacterial strains may be pathogenic or multidrug-resistant.

Additionally, the potential for multiplexing is evident: by adapting the wavelength of the light source and the detection algorithm, the system could be tuned to monitor various chromogenic or pH-sensitive media for other pathogens. The modularity of the design also makes it compatible with integration into existing laboratory incubators or portable microcontrollers for remote data collection, as suggested by Luka et al. (2017) [41].

In summary, this optical detection system achieves the following:

- Outperforms standard methods by reducing detection time from 24 h to under 3 h [37,38];
- Minimizes resource consumption by reducing agar volume from 20–40 mL to 250 µL [15];
- Avoids the need for complex instruments like PCR thermocyclers or spectrometers [7,9,38];
- Simplifies user operation, with a plug-and-play detection module and sealed sample handling [35];
- Facilitates mass adoption through scalability, cost-efficiency, and minimal training requirements [41].

Nevertheless, certain limitations remain. While the color change in ASM is highly indicative of *Staphylococcus aureus*, it is not exclusive, and false positives may occur with other mannitol-fermenting organisms [37,40]. Further refinement, such as wavelength discrimination or biochemical confirmation, could enhance specificity. Additionally, environmental conditions such as ambient light or temperature could influence measurements and should be controlled or compensated for in future iterations.

Ultimately, the presented results affirm that optical sensing coupled with colorimetric media offers a robust, scalable, and accessible alternative to both traditional culture and high-tech biosensor systems. It is a solution well aligned with the priorities of decentralized diagnostics, global health equity, and sustainable biomedical technology.

## 5. Discussion

The findings of this research validate the efficiency, practicality, and cost-effectiveness of the proposed optical sensor for detecting *Staphylococcus aureus* in mannitol salt agar (ASM). In contrast to high-complexity biosensors relying on molecular recognition elements like antibodies or nucleic acids [7,9], this sensor employs a straightforward physical principle—color change detection—that can be monitored optically. This simplicity translates into several key advantages:

First, the use of standard ASM media and conventional inoculation techniques ensures that laboratory personnel require no additional training to implement the sensor. This is in stark contrast with biosensors that demand sterile microfluidics, antibody conjugation, or microfabricated surfaces [14,36]. The ability to use traditional microbiological methods while integrating an optical readout streamlines the adoption process in clinical and field settings.

Second, the reduction of reagents by a factor of 125 represents a significant leap in sustainability and resource management. Traditional Petri dish cultures often use 30–40 mL of medium per test. The miniaturized system uses only 250 µL, which not only conserves reagents but also decreases waste generation and facilitates batch testing in small-volume formats such as microtubes or vials. This aligns with trends toward minimal sample and reagent consumption in modern diagnostic devices [15,39].

Third, time-to-detection is critically improved. The proposed prototype is not the fastest in terms of detection time since there are detection models functionalized with silver nanoparticles that have detection times as low as 20 min [27]. However, standard agar-based tests may require overnight incubation to visualize bacterial colonies and color changes, typically 18–24 h.

The sensor proposed in this study achieves detection in 150 min or less, a time reduction of over 10-fold. This is comparable to or better than some real-time imaging techniques and certainly superior to most culture-based diagnostics. In outbreak scenarios or settings where rapid response is essential, this time saving could be decisive in initiating treatment or containment measures [38,41].

Fourth, the device’s architecture is minimalistic yet functional. Comprising an LED light source optimized for red wavelengths, a light-dependent resistor (LDR), and a basic electronic circuit, the system delivers binary optical outputs (signal/no signal). Unlike systems that require spectral analysis, digital imaging, or AI-driven classification [39], this sensor provides immediate and interpretable results. Such simplicity enhances durability, reduces maintenance, and facilitates deployment in decentralized or mobile testing units.

Compared to other optical sensors, such as fiber optic configurations [14], colorimetric cameras [41], and plasmonic biosensors [37], our device is cheaper, easier to build, and simpler to integrate with standard lab workflows. These characteristics make it an excellent candidate for scalable manufacturing and distribution, especially in low-resource regions.

Concerning the spectral resolution, in the proposed system, the target signal is a broad colorimetric shift of phenol red in ASM; therefore, the relevant information is captured with discrete bands between 530 and 650 nm. In inoculated samples, the four channels show the same pattern (initial decrease, 60–110 min plateau, increase with a peak at ~150 min), whereas controls remain stable (see Figure 4 and Figure 5). Thus, increasing spectral resolution with a spectrometer (continuous spectrum) does not improve time-to-detection over a discrete multichannel readout, because the useful contrast is dominated by the global color shift rather than fine spectral features (see Figure 2, Figure 4, Figure 5 and Figure 6).

According to Table 1, LED–photodiode/LDR architectures offer the lowest cost per channel and high portability (e.g., ~USD 150 estimated) compared with spectrometer-based transmittance (~USD 300) or RGB+AI (~USD 500), which requires more complex optics/illumination and calibration yet does not shorten time-to-result in this colorimetric assay (Figure 7; LED–LDR proposal). The drawback is the lack of a continuous spectrum; as a compromise, the number of LED channels can be increased (e.g., 6–8 within 530–650 nm) to gain selectivity while maintaining low cost, reserving the spectrometer for multiplex scenarios or when deconvolution of overlapping indicators is needed.

Furthermore, the design supports future optimization. Integrating the sensor with wireless transmission modules or embedding it in automated incubators could support continuous monitoring. Multiplexing the sensor in array formats could enable parallel testing for multiple pathogens. Even the incorporation of smartphone-based light analysis, as suggested in the emerging literature [41], could add a layer of digital validation without compromising simplicity.

This study only considers the interaction of *Staphylococcus aureus* in ASM. However, we cannot ignore the fact that *S. saprophyticus* produces a similar effect in ASM but differs in its mannitol fermentation dynamics. It is possible to characterize these dynamics and establish differences in time, as well as other optical differences that may arise. Similarly, for a culture broth made for *S. saprophyticus*, we can take advantage of its resistance to novobiocin to block any false positives due to *Staphylococcus aureus* [42]. To prepare specific culture broths for each bacterium, dynamic characterizations can be performed in different agar media, and inhibitors can be used to select the type of bacteria to be detected in the environment in which they are present.

The development of optical bacterial sensors represents a tangible opportunity to improve outbreak response, monitor water and food quality, and rapidly detect clinical infections. Applications range from disposable devices for surface analysis to integrated platforms for production lines or intensive care units [20,21,22,23].

Current challenges include improving selectivity against interferences, improving the stability of optically active materials, achieving electronic miniaturization for automated reading and interpretation, and achieving clinical or regulatory validation for widespread use. The field is evolving toward hybrid systems that combine optical techniques with microfluidics, electrochemical biosensors, electrical signals, multiplexed biosensors, nanotechnology, and autonomous systems connected to mobile devices or IoT networks [19,24]. Biodegradable sensors and the integration of artificial intelligence promise increased specificity and cost-effectiveness [7,39,41].

## 6. Conclusions

This work presents an optical detection system that redefines practical microbial diagnostics by uniting conventional microbiology with accessible sensing technology. It offers a high-impact tool for applications where speed, cost, and usability are essential, without compromising diagnostic accuracy. The approach also opens pathways for future sensor innovation, particularly in alignment with global health goals for decentralizing diagnostic tools and promoting sustainable laboratory practices.

The stability of the culture broth in the prototype extends up to 6 months due to the hermetic sealing system, which maintains the appropriate conditions for bacterial growth for longer. The culture medium and the test method showed excellent repeatability, as the spectral results were always the same during the six-month experiment, regardless of the storage time of the culture broth.

The most relevant applications for this type of sensor can be summarized as follows:

- Clinical diagnostics: Early detection in hospitals to prevent nosocomial infections.
- Food industry: Online contamination monitoring in processing environments.
- Environmental monitoring: Field-deployable sensors for water quality analysis.
- Telemedicine: Optical readers integrated into smartphones for remote diagnosis

Nevertheless, certain limitations remain. While the color change in ASM is highly indicative of *Staphylococcus aureus*, it is not exclusive, and false positives may occur with other mannitol-fermenting organisms. Further refinement, such as wavelength discrimination or biochemical confirmation, could enhance specificity. Additionally, environmental conditions such as ambient light or temperature could influence measurements and should be controlled or compensated for in future iterations.

Finally, the presented results affirm that optical sensing coupled with colorimetric media offers a robust, scalable, and accessible alternative to both traditional culture and high-tech biosensor systems. It is a solution well aligned with the priorities of decentralized diagnostics, global health equity, and sustainable biomedical technology. Also, future directions to explore include multiplexed detection platforms using chromogenic media for other pathogens, the incorporation of smartphone-based analysis, and integration with IoT-enabled diagnostic units.

## Figures and Tables

**Table 1:** Comparison of optical bacteria detection sensors and their different technologies.

Type of Sensor	Detection Time (min)	Detection Limit (CFU/mL)	Estimated Cost (USD)	Portability
Transmittance	150	1000.0	300	Medium
RGB + AI	90	500.0	500	High
Optical Fiber	120	100.0	700	Medium
LED Photodiode	100	1000.0	150	High

## Data Availability

The original contributions presented in this study are included in the article, and further inquiries can be directed to the corresponding authors.

## Abbreviations

The following abbreviations are used in this manuscript:

CFU	Colony-Forming Units
RGB	Red Green Blue
Ai	Artificial Intelligence
IoT	Internet of Things
ASM	Agar Salt Mannitol
SPR	Superficial Plasmonic Resonance
LED	Light-Emitting Diode
LDR	Light-Dependent Resistor
PCR	Polymerase Chain Reaction
NAAT	Nucleic Acid Amplification Test
TSA	Tryptic Soy Agar
ROI	Region of Interest
